# Density resistance evaluation of maize varieties through new *“Density–Yield Model*” and quantification of varietal response to gradual planting density pressure

**DOI:** 10.1038/s41598-018-35275-w

**Published:** 2018-11-23

**Authors:** Liyuan Tang, Wei Ma, Mehmood Ali Noor, Lianlu Li, Haipeng Hou, Xiangyun Zhang, Ming Zhao

**Affiliations:** 1 Institute of Crop Sciences, Chinese Academy of Agricultural Sciences, Key Laboratory of Crop Physiology and Ecology, Ministry of Agriculture, Beijing, 100081 China; 2 Institute of Cotton, Hebei Academy of Agriculture and Forestry Sciences, Shijiazhuang, 050051 China; 3 College of Agronomy and Biotechnology, China Agricultural University, Beijing, 100193 China

## Abstract

Increasing planting density is the main method and key management to enhance the grain yield. Preventing lodging and premature senescence in high planting density, and screening and enhancing the density-tolerance of maize variety is the main goal of agronomy. Differential response of maize hybrids to high plant density greatly affect the dry matter accumulation and its allocation to maize kernel, depending upon various traits responsible for crowding stress tolerance, of which ear characteristics are pivotal. Density resistance as a quality appraisal of certain variety permits the construction of a simple and accurate method to determine this value, useful for plant breeding. Therefore, we created a new quantitative method, which tested several maize varieties planted populary in China (e.g. Zhengdan 958, Xianyu 335, and Denghai 661) to quantify their response to crowding stress through model. We established 13 planting densities (ranging 1.67–16.67 plants m^−2^) by adopting fixed line spacing (80 × 40 cm) and then gradually increasing row spacing from 1 m to increasing planting density. A conventional standard plot was also established for verification and evaluation of the plant morphologic characteristics, ear traits, and the yield of maize at various standard densities during 5-year study period. By studying the density–yield relationship, a quantitative model was constructed to identify the density resistance of maize. Grain yield of maize varieties under varying planting densities were simulated, and models of population yield and yield per plant that fitted the data well with high biological significance were produced. From the models, the optimal density of the popular main maize varieties planted in China and the morphological characteristics of each variety at that density were identified. The density-resistance of each variety was referred to as the ear-sensitivity classification. With the highest yield at the optimal density, the plant height of each variety reached 98% to that of tallest plant. The ear/plant ratio was about 0.45, and the ratio between the stem diameter and the largest stem diameter was 0.65–0.80. During the harvest period, the ratio between average single-plant yield and the highest single-plant yield was 0.40–0.50. By gradually increasing planting density, the density resistance of the maize and the changes in yield with density were quantified. Present study provides a convenient tool for the effective selection of varieties by plant breeders through this method and model will help to rapidly identify the density resistance for a new variety and accurate confirmation to optimal planting density, it could be optimized to enable practical production at reasonable planting densities.

## Introduction

A gigantic increase of six-fold was observed in maize grain yields since the onset of hybrid era starting from 1939 to date, mainly attributed to successful breeding strategies and superior agronomic managements, amongst which the increased planting density is a prominent contributor^1–3^. Increase in maize yield during this period is primarily due to increase in plants per unit area as a function of high planting density as compared to increase in land under maize. Linear increments in grain yield can be obtained upto a certain increase in plant population, after that this trend becomes curvilinear. The explaining factors for this curvilinear trend is the continuous resource competition between highly dense crop plants for light, water and nutrients, thus overall suppressing plant biomass and its allocation to maize grain^4^. To optimize the planting density over a given area will mainly depend upon the genetic architecture, climate adaptability, available soil moisture, soil nutrient stock, hybrid duration and adjusted row spacing for maximum light penetration^4–6^.

Crowding stress in plants is a persistent stress, continuously affecting crop plants throughout crop cycle^3^. Among crops of same family, maize is more vulnerable to be affected by varying plant population densities due to its low tillering ability^6,7^. Continuous increase in plant density however changed a number of morphological traits, e.g. more upright leafs, more light interception, reduced tassel size, short anthesis-silking interval, increased kernel weight due to prolonged grain-filling stage and more leaf area over land area^1^. The competition for resources actually starts around the flowering stage, which is critical to determine final grain yields^8^. Grain numbers per plant and per unit area and the final grain weight are considered to be the most affected yield traits due to crowding stress^6,9–11^. As proposed by Tollenaar *et al*.^12^, the maize plant accumulates its half of the total dry matter after the flowering stage, which is mainly driven by maximum light interception by additional leaf area per land area as a function of canopy photosynthesis. Another important feature to this yield increment is the genetic gain through rigorous breeding strategy of increasing yield per plant by selecting hybrids of functional stay-green and with improved source-sink balance^2,13^. Therefore, long-standing population stress during the hybrid development has additionally resulted in enhanced tolerance to various abiotic stresses^14,15^. Whereas, the yield gain due to increased planting density is associated with enhanced dry matter accumulation per unit area, which has significantly reduced the harvest index of maize crop^3,10,16^.

Although crowding stress hinders to attain full genetic potential for maize crop, but progressive breeding through successful selection under dense environments has resulted in development of maize hybrids in China which can perform best under stressful environments^10,17,18^. This genetic gain is associated with increased kernel number, improved post-silking biomass accumulation and better partitioning of attained dry matter to economic plant parts^10,18,19^. Unavoidable losses due to crowding stress, including delayed anthesis-silking interval, low kernel setting, barrenness and the prevailing abiotic stresses, will however be the tradeoffs for high yield in maize.

In recent years, maize yields in China have greatly increased. In 2015, the national maize yield was 208.12 million tons, an increase of 15.34 million tons from the previous year, mainly attributed to the increased plant density. However, as a limitation to harvest more yields, cultivating density-resistant varieties and identifying optimal densities are crucial to utilize the full genetic potential of these varieties. To elucidate the density-resistance properties of maize varieties requires studies of the relationship between density and yield. This relationship was earlier summarized using three models (equal difference, equal ratio, and mixture), and thus established the corresponding theoretical formulas, demonstrating that equal ratio model (*y* = *axe*^*−bx*^) can be applied to most of the maize varieties^20^. However, most studies from that time used spreading-leaf varieties, thus limiting the research. The relationship between yield and density was studied by using upright-leaf varieties, and the results indicated that single-plant and population grain yields both change predictably with increasing density, and that the population grain yields of upright-leaf varieties also conform to the model *y* = *axe*^*−bx*^ (a > 0, b < 0)^21^. The relationship between yield and density of different maize varieties follows a trend that can be described by a parabola, i.e., *y* = *ax*^2^ + *bx* + *c*, where *y* is yield and *x* is density^22^. The density-resistance index and marginal effect are the two major methods currently used in China to evaluate the density resistance of maize^23–25^.

However, planting multiple varieties of maize requires a large area in agronomic experiments, and no convenient system has been established yet to evaluate density resistance in smaller fields using a simple and quantitative evaluation index, which has never been addressed earlier to best of our knowledge. Keeping in view the significance of differential varietal response to increasing planting densities, we evaluated several Chinese maize hybrids over a period of five years for their characteristic and quantitative response to a range of fixed line densities. Our hypothesis states that the contradiction exists between individual plants within a plant group under conditions with limited resources, whereby if the density increases, the growth and development of an individual maize plant will be restricted, which produces a density effect. The primary objective was to characterize the studied varieties for their yield response to gradual increase in plant density, and secondly to quantify that response for the yield contributing traits through a simulation model. The motivation for this study was the urgent need to devise a method that can identify density resistance and accurately evaluate multiple crop varieties in a time- and energy-efficient manner.

## Materials and Methods

### Experimental site and maize cultivars

The study was conducted from 2007 to 2011 in the Science and Technology Demonstration Garden of the Chinese Academy of Agricultural Sciences in Langfang, Hebei Province, China (116°23′N, 39°07′E; 9 m altitude). The soil is sandy with a pH of 7.64, organic matter content of 0.62%, total nitrogen content of 0.06%, 46.3 mg kg^*−*1^ available nitrogen, 16.2 mg kg^*−*1^ rapidly available phosphorus, and 62.5 mg kg^*−*1^ rapidly available potassium.

The varieties used for testing were upright-leaf maize varieties including Xianyu 335 (XY335), Zhengdan 958 (ZD958), Denghai 661 (DH661), and spreading-leaf maize varieties including Zhongdan 808 (ZD808) and Yinong 103 (YN103). The detailed variety selection and cultivation of varieties for each experimental year is presented in Table 1.

**Table 1:** Maize varieties used from 2007 to 2011 for the quantification of planting density stress.

Year	Cultivars
2007	Zhengdan 958, Jingdan 28, Denghai 661, Denghai 701, 970
2008	Zhengdan 958, Zhongshi 839, Denghai 661, NH 0739, Xianyu 335, Zhongdan 808
2009	Zhongdan 808, Zhongshi 839, Zhengdan 958, Denghai 661, Yinong 103, Xianyu 335, Zhongdan 909, Jinke 518, 3862, 2413, 1517, 1340, 1322, 1227, 475, 250, 65
2010	Zhengdan 958, Xianyu 335, Denghai 661, Yinong 103, Zhongdan 808, Zhongdan 909, 970, 777, 972, 1048, 1443, 1461, 1185
2011	Zhengdan 958, Xianyu 335, Denghai 661, Yinong 103, Zhongdan 909, Zhengdan 18, Xuntian 16, Xundan 20, Beiqing 210

### Experimental Design and Treatment setup

**Figure 1:**
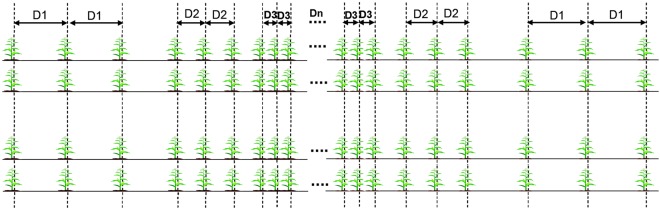
Schematics of new method of the plot with planting maize to increase the planting density gradually.

In this study, the planting density was gradually increased to identify the density resistance of various maize varieties. This was considered a quantitative analytical method that examined inward density growth, where the row spacing from the observation road, heading along the seeding ridge, narrowed toward the interior of the crop (Fig. 1). Under this continuous increase in planting density, a gradient change from low to high density was created. By investigating the major characters of the plants in response to the change in the density gradient, a dynamic model of plant characteristics was established and then used to determine the density-resistance yields of the different varieties and to quantify the density resistance and optimal planting density of each variety.

Each variety was planted in eight lines, with line spacing decreasing from 80 cm to 40 cm. Different row spacing were set for each line, with 13 row spaces set in total (100, 80, 60, 50, 45, 40, 35, 30, 25, 20, 15, 12.5, and 10 cm). The corresponding plant densities were 1.67, 2.08, 2.78, 3.33, 3.70, 4.17, 4.76, 5.56, 6.67, 8.33, 11.11, 13.3, and 16.67 plants m^−2^, respectively. Five seeds were planted for each density tested, with the row spacing decreasing after every five rows from the observation road into the plot, gradually increasing the density gradient. Different varieties were planted symmetrically from the low-density region at the side of the plot to the central high-density area. Varieties of similar phenotypes (i.e., plant height and plant type) were planted adjacent to each other.

For each test area, a final thinning was conducted during the trefoil stage to attain the required density. Before planting the seeds, the moist soil in the field was carefully prepared through recommended ploughings, throughout the study period (5 yr). The base fertilizers applied at recommended rates included nitrogen (22.5 g m^*−*2^), P_2_O_5_ (17.3 g m^*−*2^), and K_2_O (15.0 g m^*−*2^), which was kept constant for each experimental year. During the tasselling stage (VT), additional split of nitrogen (13.8 g m^*−*2^) was applied as side-dressing at sufficient soil moisture content. Cultivation and agronomic management measures such as regular watering, timely weed control, and insect and disease prevention were regularly undertaken for whole study period, and the plots were generally managed to the same standard as a high-yield maize fields.

### Density Verification test

A field-density verification test was undertaken during the whole study period (5 yr). For this purpose a standard plot was established for verification and evaluation of morphological characteristics, ear traits, and the yield of maize at gradually increasing densities during whole period. The yield data of this test field, where traditional planting standards were adopted, was used for verification. Each variety under this verification test was grown at four densities (4.5 plants m^*−*2^, 6.0 plants m^*−*2^, 7.5 plants m^*−*2^, and 9.0 plants m^*−*2^) in 132 m^2^ (27.5 m × 4.8 m) plots, with 3 replicates. Each plot contained eight lines with planting spacing of 80 cm × 40 cm.

### Crop Measurements and Methods

Standard staging system^26^ was used during the test period to identify the development stages of maize varieties, and a stage was characterized when 50% of plant population reached the corresponding stage, viz. V6, V12, VT, R3 and R6. At physiological maturity, during the final harvest bare-plant percentage (barrenness) was recorded for each population.

For yield determination, four random rows for each plot of specific variety at each density level were selected, corresponding to 12 plants as sample size. Prior to harvest, ears per plant (prolific ears) were counted for each plot to calculate number of ears per unit area. After that plants were manually cut at the ground level and yield parameters, requiring immediate measurements, were recorded. Grain yield per plant was estimated and then population yields were computed. All the cobs were harvested and yield attributes viz. ear length, bald-tip length, ear perimeter, ear number per unit area, grain number per unit area and thousand kernel weight were measured for each harvested unit plot during each experimental year (2007–2011). Grain yield was calculated and presented at a moisture content of 14% after measuring moisture content of the harvested kernels with a PM-8188 new grain moisture meter (Kett Electric Laboratory, Tokyo, Japan). The same yield and morphological parameters were also recorded for standard field-density verification test plot over the study period as for gradually increasing density plots, whereas, the additional measurements done were viz. plant height, ear height, ear to plant height ratio, stem diameter, grain yields and dry matter accumulation per plant organ, following standard procedures.

#### Correlation analysis

The dynamic relationship between the yield of a single plant and planting density was simulated using Curve Expert 1.38. The first 10 simulation equations that produced good simulation results were used to select a simulation model (Weibull model) that had biological plausibility and correctly reflected the changes in yield of a single plant at different planting densities (Table 2). The equation of the model was ${\rm{y}}={\rm{m}}-{{\rm{ne}}}^{(-{{\rm{px}}}^{{\rm{t}}})}$, having significant coefficient values (*r* = 0.9422**).

**Table 2:** Simulated curve parameters for density-relative single plant yield for maize.

Model	Parameter				SD	*R* ^2^
	a	b	c	d		
y = ab^(1/x)^x^c^	6.3283	0.1224	−1.0341		0.0778	0.9425**
y = e^(a+b/x+clnx)^	1.8450	−2.1002	−1.0341		0.0778	0.9425**
y = (a + bx)/(1 + cx + dx^2^)	−55.4524	68.1952	13.0568	11.8894	0.0779	0.9426**
${\rm{y}}={\rm{a}}-{{\rm{be}}}^{(-{{\rm{cx}}}^{{\rm{d}}})}$	1.0896	0.9565	11.4042	−1.4205	0.0781	0.9422**
y = a + bx + cx^2^ + dx^3^	1.3766	−0.1462	0.0063	−0.0001	0.0791	0.9407**
y = a + bx + cx^2^	1.3349	−0.1275	0.0040		0.0792	0.9405**
y = a + b*cos*(cx + d)	2.8548	2.5391	0.0584	2.2226	0.0794	0.9403**
y = 1/(a + bx^c^)	0.7559	0.0544	1.3890		0.0795	0.9399**
y = 1/(a + bx + cx^2^)	0.6892	0.0922	0.0046		0.0801	0.9390**
y = (a + bx)^(−1/c)^	0.8241	0.0596	0.4824		0.0802	0.9389**

*x* and *y* in the model denote density and relative single-plant yields, respectively. **Significant at the 0.01 probability level.

The population yield data for several representative maize varieties at different planting densities were used to derive an equation for simulations (Table 3). The Weibull model had the best predictive effect. The parameters for the various varieties differed, but the coefficients of association were all > 0.99.

**Table 3:** Equations for simulating grain yields at different planting densities.

Model	Parameter				SD	*R* ^2^
	a	b	c	d		
y = a + bx + cx^2^ + dx^3^	0.0866	0.3795	−0.0366	0.0011	0.1617	0.7094**
y = (a + bx)/(1 + cx + dx^2^)	−0.0750	0.4998	0.1251	0.0155	0.1625	0.7059**
y = ab^x^x^c^	0.5139	0.9142	0.8004		0.1642	0.6978**
y = ab^(1/x)^x	4.4996	0.0375	−0.4096		0.1653	0.6921**
y = e^(a + b/x + cln(x))^	1.5039	−3.2825	−0.4096		0.1653	0.6921**
$y=a-{{\rm{be}}}^{(-{{\rm{cx}}}^{{\rm{d}}})}$	1.2547	0.8139	0.0876	2.1365	0.1690	0.6766**
y = a/(1 + e^(b−cx)^(1/d)^)	1.2801	5.2384	1.3602	4.3607	0.1744	0.6485**
y = a/(1 + be^−cx^)	1.2842	5.6329	0.9126		0.1748	0.6396**
y = (ab + cx^d^)/(b + x^d^)	0.6216	290.2861	1.2823	4.9440	0.1754	0.6389**
${\rm{y}}={\rm{a}}{{\rm{e}}}^{-{{\rm{e}}}^{{\rm{b}}-{\rm{c}}{\rm{x}}}}$	1.2858	1.1270	0.7789		0.1754	0.6338**
y = a(b − e^−cx^)	2.0265	0.6320	0.6188		0.1711	0.6299**
y = a + bx + cx^2^	0.5760	0.1534	−0.0073		0.1728	0.6266**
y = a + b*cos*(cx + d)	−7.4411	8.8201	0.0401		0.1732	0.6245**
y = a(1−e^−bx^)	1.2943	0.4721			0.1737	0.6231**
y = a${{\rm{e}}}^{(-({\rm{x}}-{\rm{b}}{)}^{2}/2{{\rm{c}}}^{2})}$	1.3785	10.4161	9.0758		0.1760	0.6224**
y = 1/(a + bx + cx^2^)	1.2646	−0.1042	0.0050		0.1787	0.6066**
y = a + b/x	1.4096	−1.2566			0.1794	0.6017**
y = ae^b/x^	1.4296	−1.0818			0.1824	0.5835**
y = ab^1/x^	1.4296	0.3390			0.1824	0.5835**
y = ax/(b + x)	1.4417	1.2856			0.1856	0.5632**

*x* and *y* in the model denote density and relative grain yield, respectively. **Significant at the 0.01 probability level.

#### Model construction

The 50 groups of different yield data for all spring maize varieties planted with increasing density in 5 yr period were processed using a normalization method by mixing the limits of the varieties. The dynamic relationship between relative population yield and planting density, produced by using Curve Expert 1.38, was used to select a dynamic curve model that had biological plausibility and accurately reflected changes in maize yield with increasing density (Table 4).

**Table 4:** Parameters used in the curve simulating density-relative single plant yield.

Cultivar	Parameter				SD	*R* ^2^
	m	n	p	t		
Xianyu 335	1.0589	0.8221	21.6819	−1.8333	0.0162	0.9985**
Denghai 661	1.2681	1.0996	6.6907	−1.2897	0.0163	0.9984**
Zhengdan 958	1.0602	0.9612	15.1895	−1.5032	0.0146	0.9988**
Yinong 103	1.1935	0.8886	25.4885	−2.6048	0.0475	0.9919**
Zhongdan 808	1.1983	0.9747	16.5011	−2.0138	0.0248	0.9985**

**Significant at the 0.01 probability level.

#### Adaptability analysis

The population yield adaptability of maize varieties under density pressure was derived from the model equation. The equation for the rate of changes in yield with increased adaptability was y = *ab*^*x*^*x*^*c* − 1^(*x* 1n *b* + *c*). When the rate of change in the population yield drops to 0, the corresponding density was considered the optimal density of that variety.

#### Model verification

To verify the accuracy of the models under actual field conditions and in traditional plant breeding, the yield data from the models were calibrated against the actual yields from standard plots with various planting densities to determine the correct planting density for each variety and the optimal planting density according to the model data. The density gradient of the standard plot was used in the model equation to obtain the simulated yield, and a regression equation was derived based on the simulated and actual yields of the standard plot.

#### Morphology response estimation

To determine the relationship between plant morphological traits in different maize varieties under density pressure and yield per unit area, and to establish the common characteristics of the plant morphological index at the highest yield, the dynamic changes between the plant morphological index and yield per unit area were simulated and compared and calibrated against the yield of a traditional standard plot.

### Data Analysis

The normalization of statistics was employed by taking yield at lowest planting density as “1”. Simulations were done for relative yield and planting density using Curve Expert 1.38 software. Then by screening of varieties against crowding stress, model of relative yield-planting density was established. The observed data were analyzed by analysis of variance (ANOVA) through statistical software SPSS version 16.0 (SPSS, Inc., Chicago, IL, USA), and the treatment means were compared thereafter. For correlation analysis, Pearson correlation coefficients were computed through SPSS too. In addition, graphing DPS (Data Processing System) v 7.05, Excel 2003 and Sigmaplot 10.0 were also used to compute, analyze and interpret the data.

## Results

### Verification of the Method using Gradually Increasing Plant Density

The difference in yields between the method that gradually increased plant density and the traditional planting was not great (Table 5). The response of plants to gradually increasing density was similar to the response observed on the standard plot.

**Table 5:** Yield comparison of Zhengdan 958 using new mthod of gradually increased density and traditional planting density.

Density (plant m^−2^)	Zhengdan 958		Deviation (%)
	Yield of the standard plot (g m^−2^)	Yield of gradually increased planting density (g m^−2^)	
4.5	855.54^a^	863.74^a^	0.96
6.7	883.19^a^	866.26^a^	−1.92
9.0	1024.18^a^	1014.01^a^	−0.99
11.2	910.99^a^	888.03^a^	−2.52

### The Density-Yield Model

#### Correlation Analysis of Density and Yield

**Figure 2:**
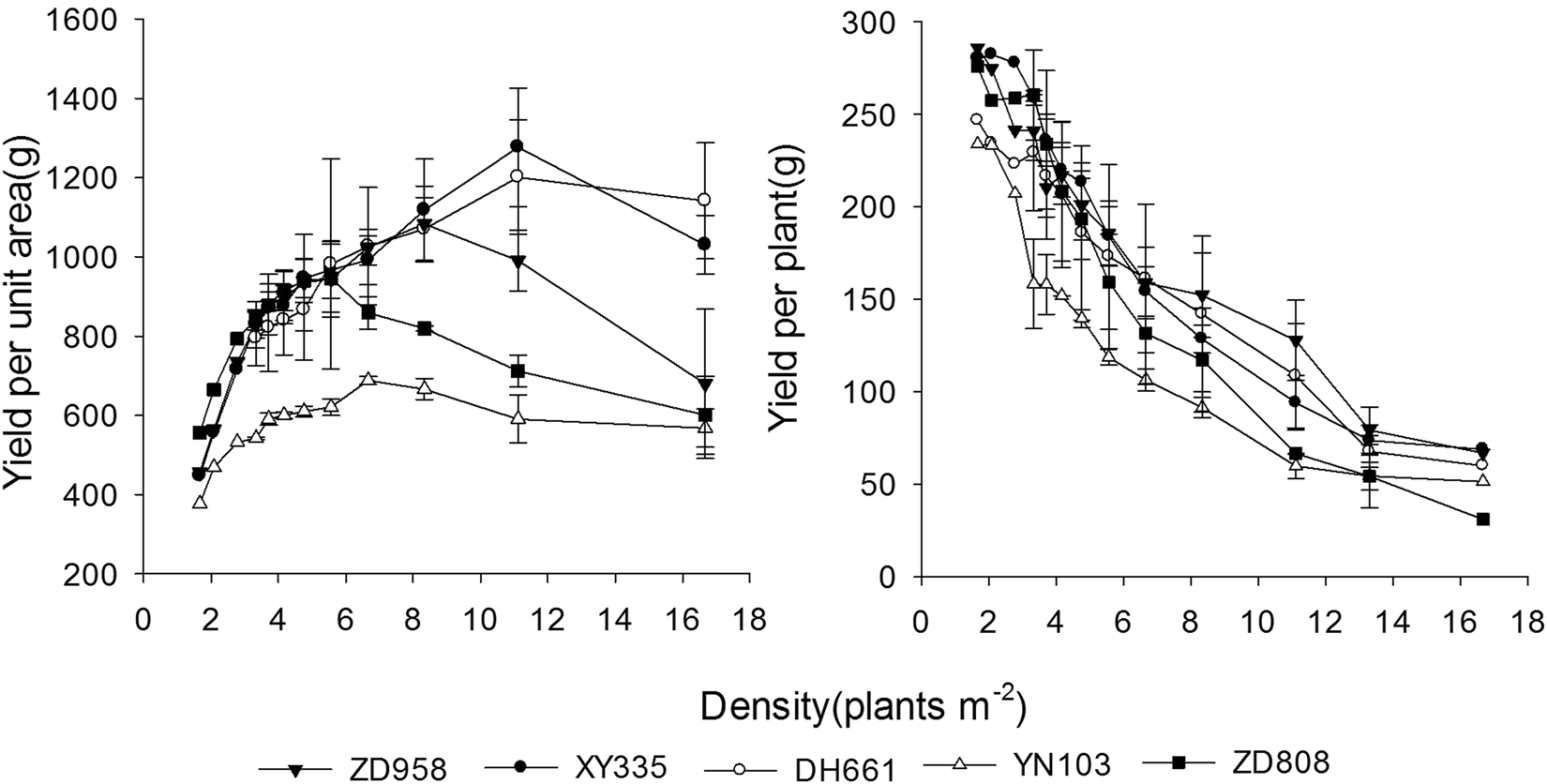
Influence of planting density on the yields of various maize varieties from year 2010 to 2011.

The yields per unit area of the various varieties and the yield of a single variety at different densities differed greatly. As shown in Fig. 2, with increasing planting density, the yield per unit area of each maize variety displayed a dynamic trend, initially rising before decreasing, while the yield of a single plant gradually declined. Over the range of densities examined in the study, the population yields of the different varieties followed the order Xianyu 335 > Denghai 661 > Zhengdan 958 > Zhongdan 808 > Yinong 103. At the high density of 11.1–16.7 plants m^*−*2^, the population yields of Xianyu 335 and Denghai 661 were still higher than those of the other varieties. The highest yields of single plants followed the order Zhengdan 958 > Xianyu 335 > Zhongdan 808 > Denghai 661 > Yinong 103.

#### Construction of the Density and Relative Yield Model

The response of the various maize varieties to the density gradient indicated that the population yield and the yields of single plants changed substantially with increased density (Fig. 2). However, the overall trend in the change in yield was consistent. If the yield for the minimum planting density (3.33 plants m^*−*2^) in 2010 was set as 1, the differences between varieties and years was removed by processing the yield data using a normalization technique. The resulting normalized data better simulated the common changes in yield with increasing plant density.

As shown in Table 5, among the simulated curve models, the best simulation effects were achieved for a polynomial fit (1), rational function (2), and Hoerl model (3). Models with biological plausibility that could accurately reflect the response of population yield to the density gradient and the three fitted equations were determined by solving their limit values:

1 $$\mathop{\mathrm{lim}}\limits_{x\to 0}{\rm{f}}({\rm{x}})=\mathop{\mathrm{lim}}\limits_{x\to 0}{\rm{a}}+{\rm{bx}}+{{\rm{cx}}}^{{\rm{2}}}+{{\rm{dx}}}^{{\rm{3}}}=a\,\mathop{\mathrm{lim}}\limits_{x\to \infty }{\rm{f}}({\rm{x}})=\mathop{\mathrm{lim}}\limits_{x\to \infty }{\rm{a}}+{\rm{bx}}+{{\rm{cx}}}^{{\rm{2}}}+{{\rm{dx}}}^{{\rm{3}}}=\infty $$

2 $$\mathop{\mathrm{lim}}\limits_{x\to 0}{\rm{f}}({\rm{x}})=\mathop{\mathrm{lim}}\limits_{x\to 0}(a+\mathrm{bx})/(1+{\rm{cx}}+{{\rm{dx}}}^{{\rm{2}}})=a\,\mathop{\mathrm{lim}}\limits_{x\to \infty }{\rm{f}}({\rm{x}})=\mathop{\mathrm{lim}}\limits_{x\to \infty }({\rm{a}}+\mathrm{bx})/(1+{\rm{cx}}+{{\rm{dx}}}^{{\rm{2}}})={\rm{0}}$$

3 $$\mathop{\mathrm{lim}}\limits_{x\to 0}{\rm{f}}({\rm{x}})=\mathop{\mathrm{lim}}\limits_{x\to 0}{{\rm{ab}}}^{{\rm{x}}}{{\rm{x}}}^{{\rm{c}}}=0\,\mathop{\mathrm{lim}}\limits_{x\to \infty }{\rm{f}}({\rm{x}})=\mathop{\mathrm{lim}}\limits_{x\to \infty }{{\rm{ab}}}^{{\rm{x}}}{{\rm{x}}}^{{\rm{c}}}=0$$

**Figure 3:**
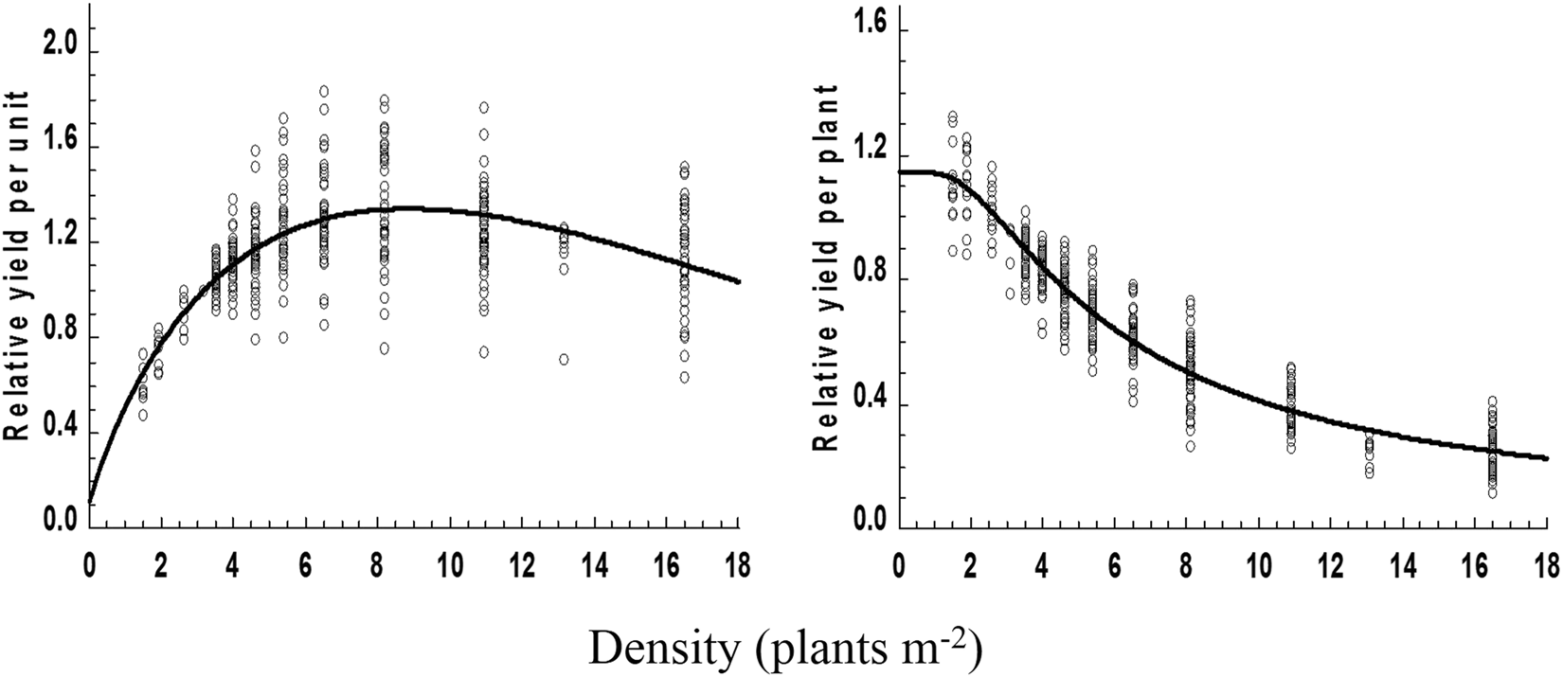
Simulated curves of the density-relative yield.

In theory, the relative population yields of maize when planted extremely sparsely and extremely densely tend to be zero. However, in equations 1 and 2, when *x* → 0, *y* → a, a is a nonzero constant, which does not conform to the population yield when the planting density is extremely sparse. Moreover, in equation 1, when *x* → ∞, *y* → ∞, which does not conform to the trend in population yields when the density increases without limit. Although the simulated correlations of Equations 1 and 2 are higher, they do not have biological plausibility. In Equation 3, 0 < a, b < 1, c > 0, when *x* → 0, *y* → 0, which indicates the population yield of maize when the planting density is extremely sparse or even zero. In addition, when the curve model parameters are (0, ∞), the equation has only one value; when *x* → ∞, *y* → 0, which indicates the dynamic change in the population yield when the density of maize increases without limit. Therefore, the curve model correctly reflected the changes in population yields for all maize varieties; the degree of fitting of the model was high and it had biological plausibility. As a result, the corresponding curve, when the Hoerl model was selected, is shown in the Fig. 3 and its equation is *y* = 0.5139*0.9142^x^*x^0.8004^ (*r* = 0.6978**).

In the model of population yield, a is the range of population yield with changes in planting density before the maximum potential yield is reached, and b is the declining rate of population yields after a maize variety reaches its maximum potential yield. When the density is −c/lnb, the highest population yield of the maize variety has been achieved, and this density is defined as the optimal density for that variety. Therefore, b and c jointly determine the optimal density and degree of density resistance, and a, b, and c jointly influence the population yield at different planting densities.

The population yield data of the main maize varieties were used to build relative-yield equations (Table 6) according to the optimal simulation curve equation. The coefficients of association of these equations were all above 0.7871**, indicating that the simulation was acceptable. The optimal density indicates the degree of density resistance in a maize variety, with higher −c/lnb values indicating greater density resistance. Density resistance followed the order Denghai 661 > Xianyu 335 > Zhengdan 958 > Yinong 103 > Zhongdan 808.

**Table 6:** Parameters used in the curve simulating density-relative grain yields for various maize varieties.

Varieties	Parameter			−c/lnb	SD	*R* ^2^
	a	b	c			
ZD958	0.5903	0.9272	0.6474	8.5623	0.0905	0.8046**
XY335	0.4921	0.9138	0.8044	8.9289	0.0622	0.9398**
DH661	0.3939	0.8937	1.0463	9.3089	0.1419	0.8362**
YN103	0.5525	0.9025	0.8249	8.0448	0.0533	0.9615**
ZD808	0.5754	0.8813	0.7797	6.1722	0.1095	0.7871**

**Significant at the 0.01 probability level.

### Density Resistance

#### Optimal Density of a Variety

Numerical values were input into the population yield simulation equation. As shown in Fig. 2, the population yields of the various varieties all increased with increased planting density, owing to differences in the potential productivity and density resistance of individual varieties, the optimal density and the highest yield of all varieties differed. The highest simulated population yields were in the order Xianyu 335 > Denghai 661 > Zhengdan 958 > Zhongdan 808 > Yinong 103, which corresponded to actual yields (Fig. 3).

#### Adaptability of the Maize Varieties under Density Pressure

**Figure 4:**
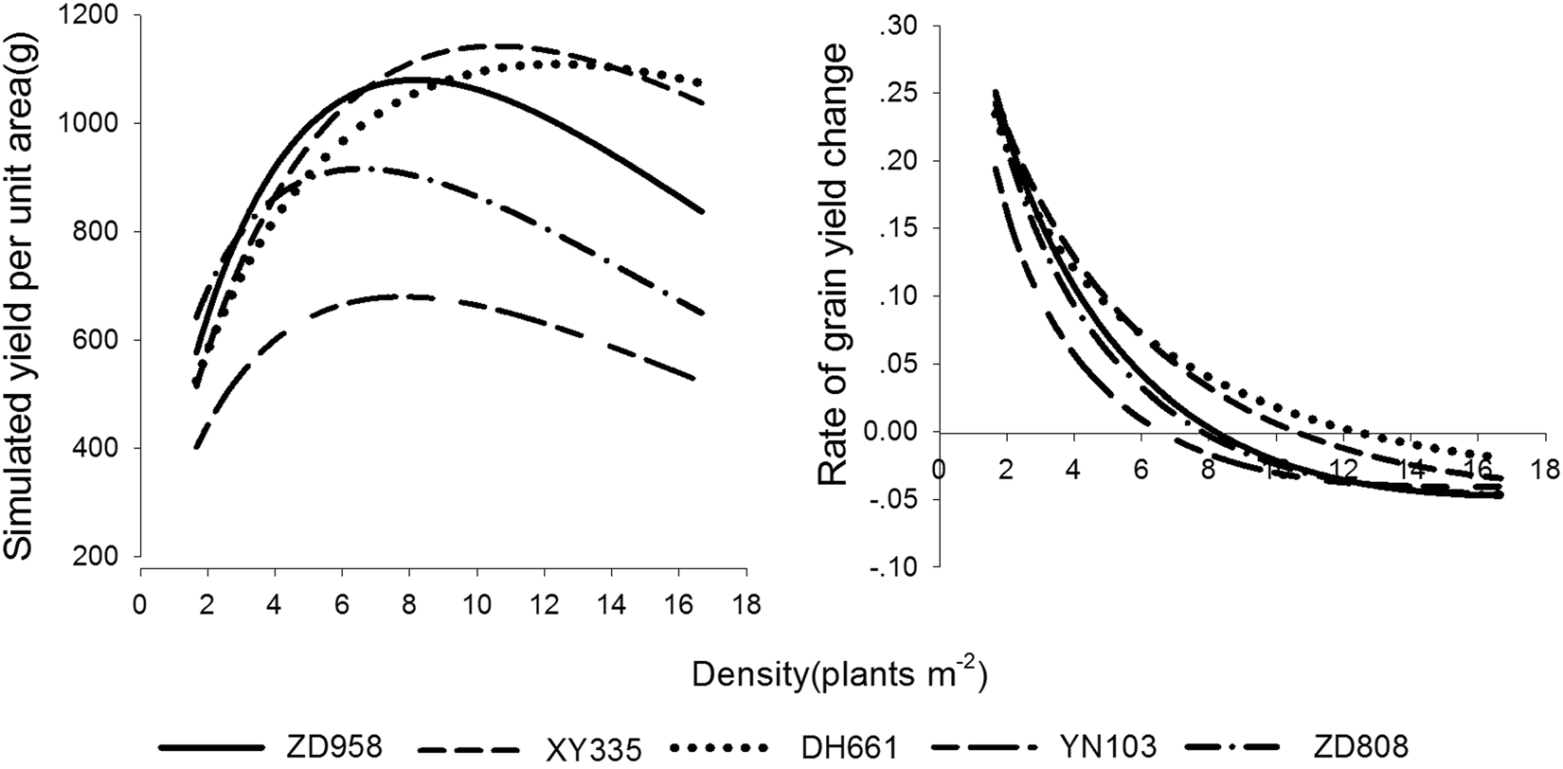
Simulated grain yields and the rates of grain yield change for various maize varieties.

As shown in Fig. 4, the rates of change in the population yields of the upright-leaf varieties of maize were slower than those of the spreading-leaf varieties. The rates of change in population yields for optimal planting densities followed the order: Zhongdan 808 > Yinong 103 > Zhengdan 958 > Xianyu 335 > Denghai 661; the higher the rate of change, the more sensitively a variety responded to planting density.

According to the analysis, the density resistance of upright-leaf maize varieties followed the order: Denghai 661 > Xianyu 335 > Zhengdan 958, while the density resistance of spreading-leaf maize varieties followed the order: Yinong 103 > Zhongdan 808. The density resistance of the three upright-leaf maize varieties was greater than that of the two spreading-leaf maize varieties.

**Figure 5:**
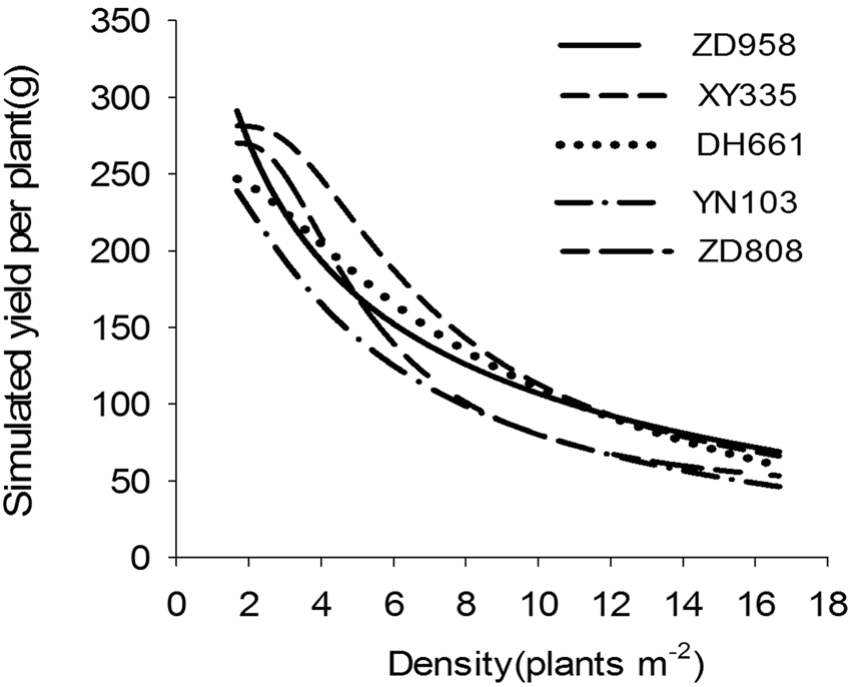
Simulated single-plant yields of different maize varieties.

With increased planting density, the yield of a single plant of each maize variety dropped (Fig. 5). The downtrend was initially slow but then increased, finally decreasing at high planting densities. The density resistance of a variety reflects the rate of decline of single-plant yields under high-density conditions. As shown in Fig. 5, the average rates of decline followed the order Zhongdan 808 > Zhengdan 958 > Yinong 103 > Xianyu 335 > Denghai 661.

When the density reached 5.0 plants m^*−*2^, the yields of Zhengdan 958, Denghai 661, and Xianyu 335 were maintained at higher levels than that of Zhongdan 808. The single-plant yield of Yinong 103 was the lowest under the same density conditions. Considering the definition of density resistance, whereby a maize variety has comparatively high single-plant yields at high densities, and the results of the analysis, the density resistance of the upright-leaf maize varieties (Xianyu 335, Denghai 661, and Zhengdan 958) was higher than that of the spreading-leaf varieties (Zhongdan 808 and Yinong 103) (Fig. 5).

### Verification of the Density-Relative Yield Model

**Figure 6:**
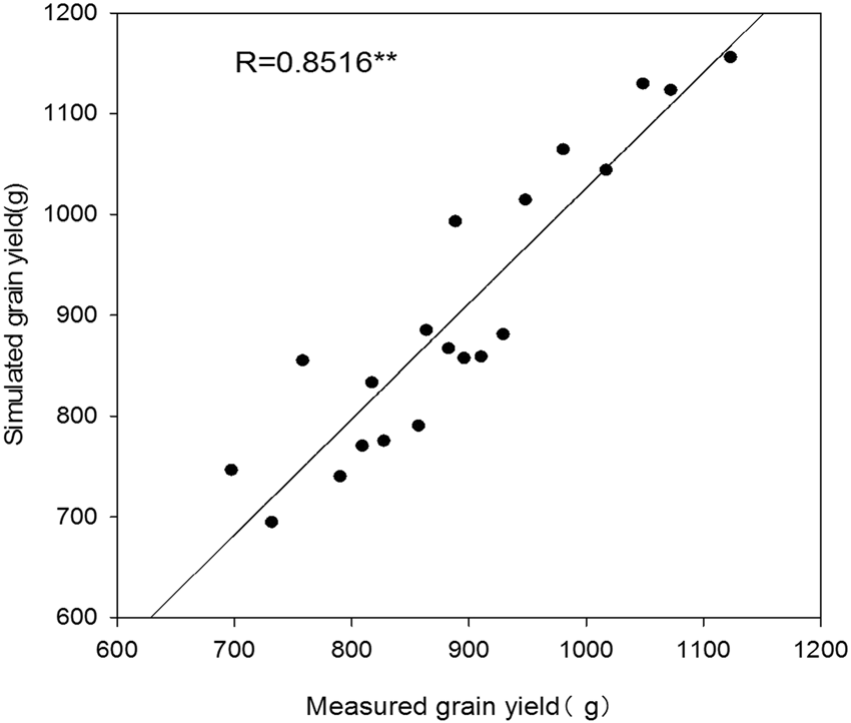
Relationship between simulated grain yields and measured grain yields of maize.

The correlation between the simulated yield for gradually increasing density and the actual yield of a standard plot was very strong and the degree of accuracy was 0.8516** (Fig. 6). A comparison of the two groups of yields for each variety indicated that the yield difference between traditional planting and using increasing planting density was not large, with the average fluctuation in yield being only 4.45%. As a result, the yield derived from the increasing density planting model can be used to represent the population yield of maize grown using the traditional method, and the model can also be used to assess density resistance in maize and to screen varieties.

### Maize Varieties’ Characteristic Response under Density Pressure

#### Ear Morphosis

**Figure 7:**
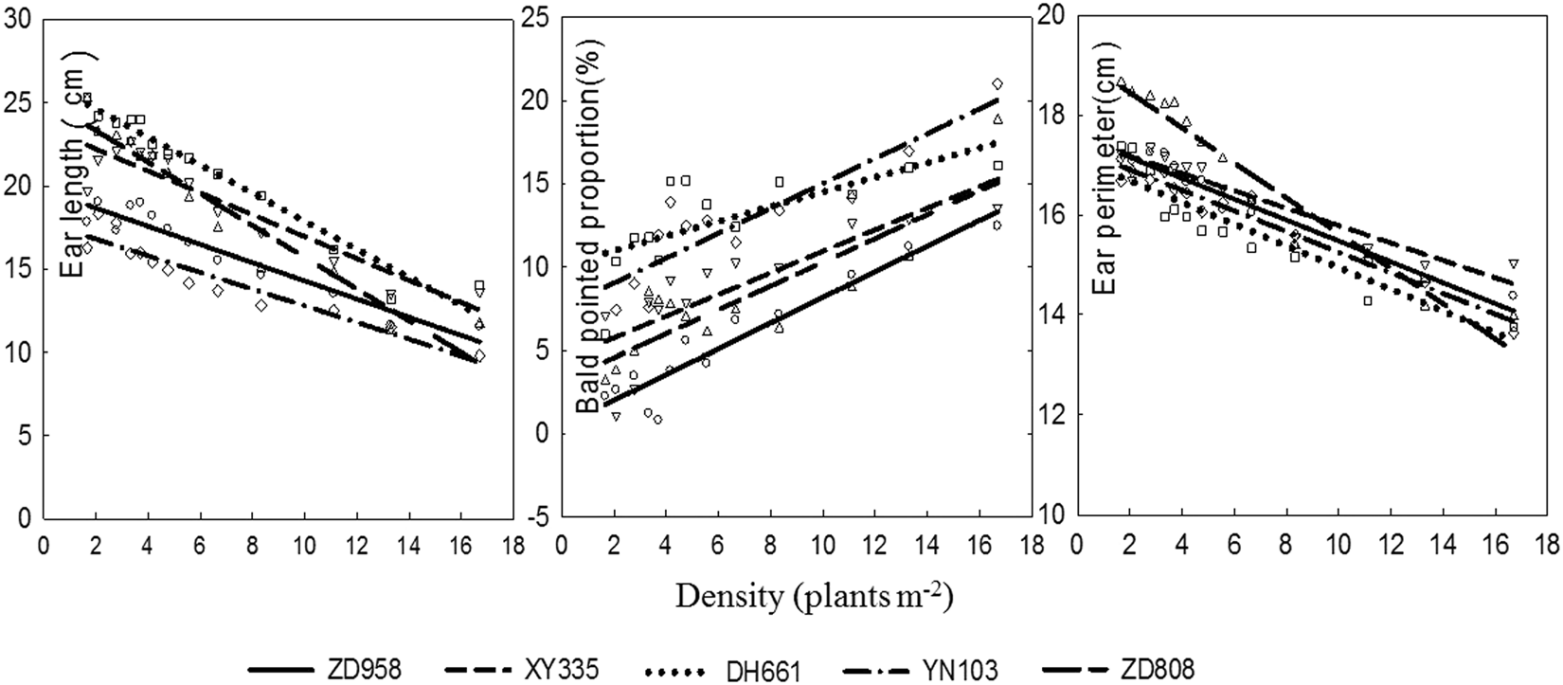
Dynamic responses of maize ears to planting density in the different varieties.

Ear length, ear perimeter, and ear bald-tip length differed significantly among plants grown at different densities. As shown in Fig. 7, with an increase in planting density, the ear morphological index changed consistently: ear length and ear perimeter decreased progressively and ear bald-tip length increased. The sensitivity of these ear traits to density differed among varieties. With increased planting density, the rates of morphological change and levels of sensitivity increased. The rates of decrease in ear length followed the order: Zhongdan 808 > Denghai 661 > Xianyu 335 > Zhengdan 958 > Yinong 103. Therefore, the ear length of Zhongdan 808 reacted to changes in planting density most sensitively, while Yinong 103 exhibited the the least sensitive response. The ear perimeter decreased in the order: Zhongdan 808 > Denghai 661 > Zhengdan 958 > Yinong 103 > Xianyu 335. Therefore, the ear perimeter of Zhongdan 808 reacted most sensitively to planting density, while Xianyu 335 exhibited the least sensitive response (Fig. 7).

#### Ear Yield Components

Under planting using gradually increasing density, the different varieties responded to changes in planting density with different intensities due to their individual traits, which affected the development of the number of ears on a single plant (see Table 7). No double ears were observed in Denghai 661 or Zhongdan 808, but Zhengdan 958, Xianyu 335, and Yinong 203 had double ears at densities of 1.7–6.7, 1.7–3.7, and 1.7–4.8 plants m^*−*2^, respectively. The rates of occurrence of double ears in the three varieties decreased quickly with increased planting density until reaching zero.

**Table 7:** Rates of occurrence (%) of double ears in the various maize varieties with gradually increasing plant densities.

Cultivar	Density (plants m^−2^)												
	1.7	2.1	2.8	3.3	3.7	4.2	4.8	5.6	6.7	8.3	11.1	13.3	16.7
ZD958	40	35	35	7.5	5	5	5	2.5	2.5	0	0	0	0
XY335	55	22.5	5.1	2.5	2.5	0	0	0	0	0	0	0	0
DH661	0	0	0	0	0	0	0	0	0	0	0	0	0
YN103	42.5	25	22.5	15	5	2.5	2.5	0	0	0	0	0	0
ZD808	0	0	0	0	0	0	0	0	0	0	0	0	0

Ear traits were further studied by classifying the different levels of double ears among the different maize varieties: Zhengdan 958, Xianyu 335, and Yinong 103 were classified into one group with low densities of double ears; Denghai 661 and Zhongdan 808 were classified into another group that had only single ear regardless of planting density.

The numbers of ears, grains per ear, and thousand-kernel weights of the two groups of maize varieties were analyzed. The results indicated that with an increase in planting density, the ear numbers for the maize varieties in both groups initially increased before later declining. The grains per ear and thousand-kernel weights of the varieties with double ears (Zhengdan 958, Xianyu 335, and Yinong 103) also initially increased before rapidly declining later. This was because the varieties with double ears had higher double-ear rates at lower planting densities due to the lower density pressure.

**Figure 8:**
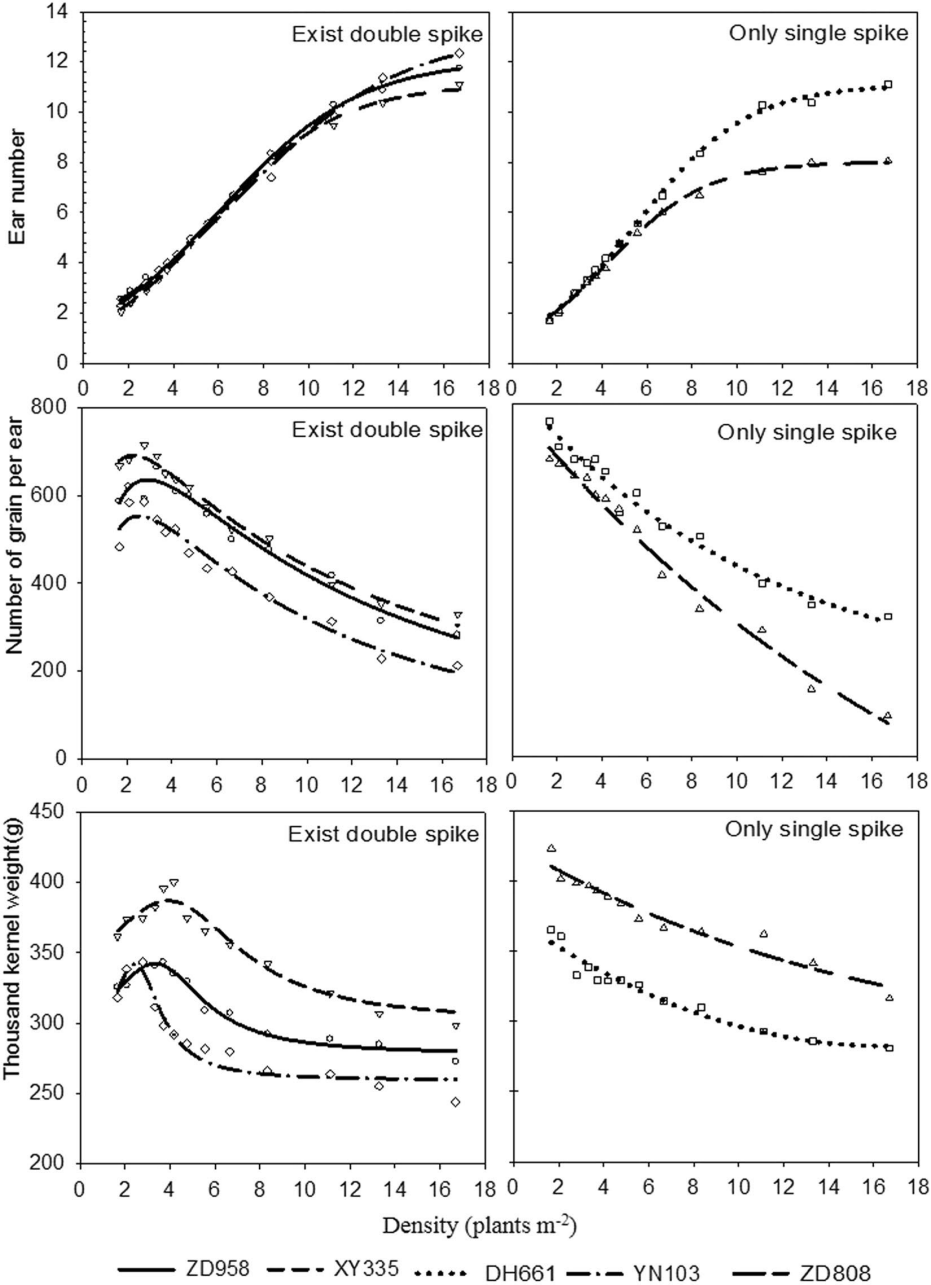
Dynamic response of ear traits to densities in different maize varieties.

With increased planting density, the grains per ear and thousand-kernel weights both decreased (Fig. 8). The smaller the synchronized range of reduction in the number of seeds per ear and thousand-kernel weight is, the slower their response to planting density was. The reduction in the range of seeds per ear of the varieties followed the order: Zhongdan 808 > Yinong 103 > Denghai 661 > Xianyu 335 > Zhengdan 958. The reduction in the range of the thousand-kernel weights of the varieties followed the order: Zhogndan 808 > Denghai 661 > Yinong 103 > Xianyu 335 > Zhengdan 958. Planting density had more impact on the number of seeds per ear than on thousand-kernel weight.

#### Analysis of Ear Traits and Yield

Ear traits, including ear length, bald-tip length, ear perimeter, ear number per unit area, grain number per unit area, thousand-kernel weight, and the yield of each variety were analyzed (Table 8). The results indicate that with gradually increasing plant density, ear length, ear perimeter, and thousand-kernel weight all declined and were significantly positively correlated with single-plant yields (*r* = >0.6833**). In contrast, the bald-tip length, ear number per unit area, and grain number per unit area all increased, with a significant negative correlation with single-plant yields (*r* = >0.5575**). Ear number per unit area and grain number were significantly positively correlated with yield per unit area (*r* = >0.4584**) while ear length, bald-tip length, and thousand-kernel weight, representing single-plant yield, were significantly correlated with the yield per unit area.

**Table 8:** Correlation analysis between ear traits and yield (*n* = 65).

Yield index	Items					
	Ear length	Bald-tip length	Ear perimeter	Ear number per unit area	Grain number per unit area	Thousand kernel weight
Ear bald-tip length	−0.4464**					
Ear perimeter	0.7470**	−0.7226**				
Ear number per unit area	0.0972	−0.0015	−0.2004			
Grain number per unit area	−0.0267	−0.1029	0.0189	0.7085**		
Thousand-kernel weight	0.7837**	−0.6147**	0.4292**	−0.6719**	−0.4277**	
Yield of single plant	0.8533**	−0.6936**	0.8620**	−0.9210**	−0.5575**	0.6833**
Yield per unit area	−0.0722	0.1449	−0.2123	0.4584**	0.8332**	−0.0800

**Significant at the 0.01 probability level;*Significant at the 0.05 probability level.

Of the ear traits that were affected by density, the length of a single-ear bald tip was significantly positively correlated (*r* = >0.4464**) with ear length, ear perimeter, and thousand-kernel weight, and ear length was significantly positively correlated (*r* = >0.7470**) with ear perimeter and thousand-kernel weight. The thousand-kernel weight was significantly correlated with each ear trait. Ear numbers per unit area of both plant groups were significantly positively correlated with grain number (*r* = 0.7085**).

### Plant Morphology Response under Density Pressure

**Figure 9:**
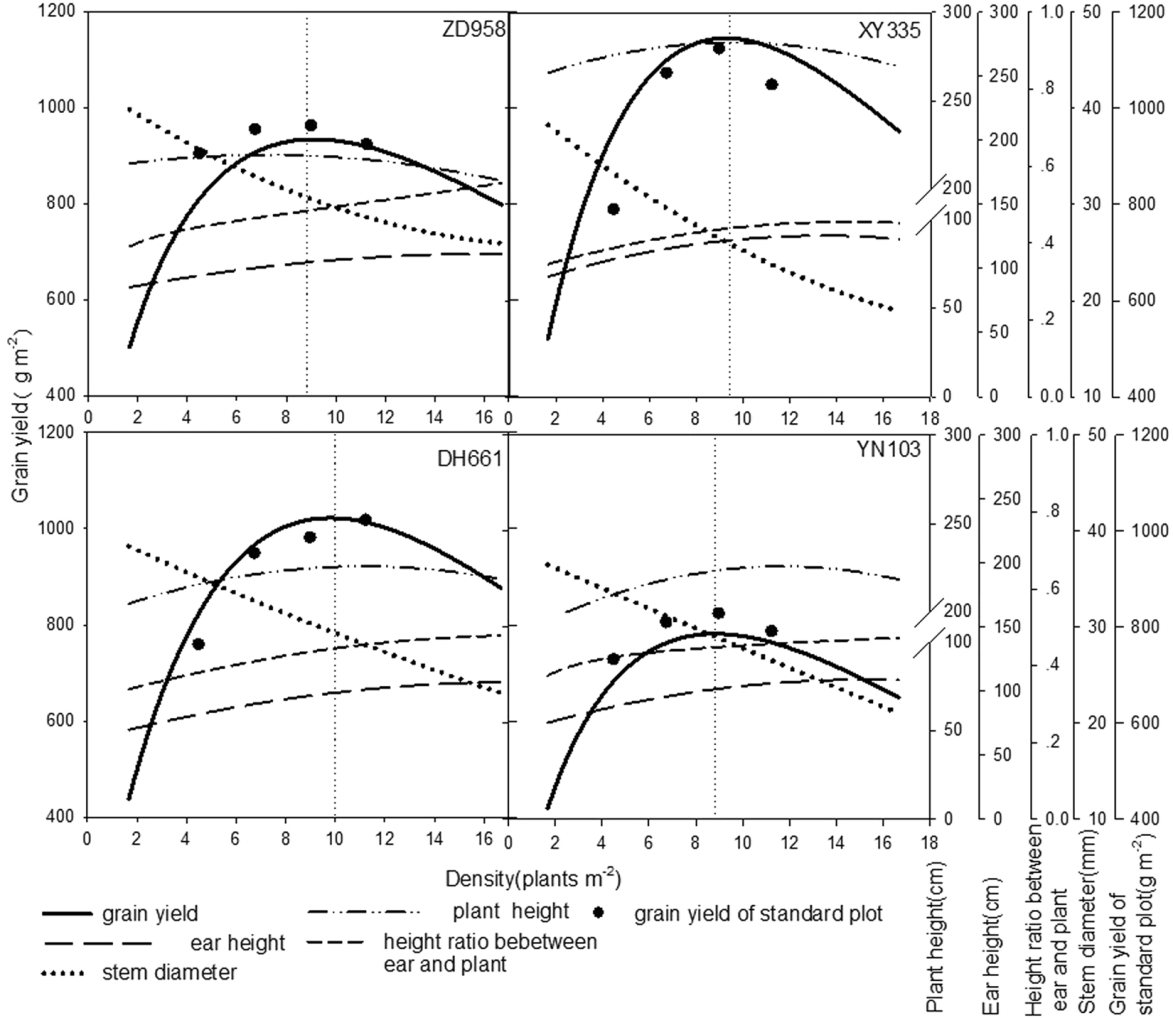
Correlations of the grain yield and the plant morphological indices for different maize varieties.

The results indicate (Fig. 9) that when the planting density was gradually increased from 1.67 plants m^*−*2^ to 16.67 plants m^*−*2^, the yield of each variety had a tendency to initially rise and later fall. The highest yield performance followed the order: Xianyu 335 > Denghai 661 > Zhengdan 958 > Yinong 103. When the highest yield was achieved, although the value of the plant morphological index for each variety was different, they had the same rate of change. The plant height of each variety reached 98.9% of the height of the tallest plant and the ear/plant ratio was between 0.43 and 0.48. The ratio between the stem parameter in florescence and the stem parameter at the lowest density was between 0.68 and 0.80.

### Research on Improving Yields by Planting Management

#### Relationship between Single-plant Yield, Group Ear Number, and Group Yield under Density Pressure

**Figure 10:**
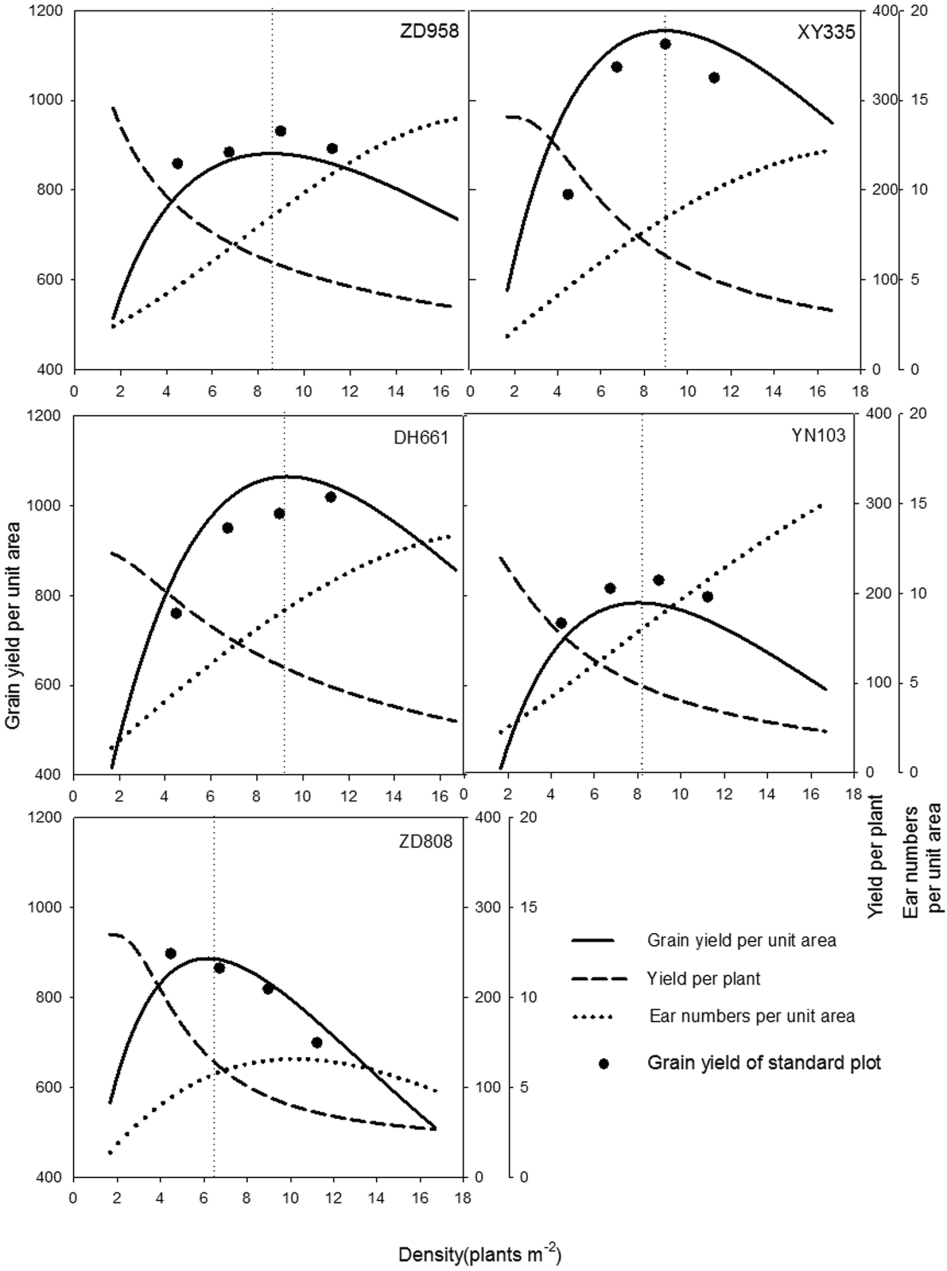
Relationships among yield per plant, ear numbers, and grain yield per unit area.

As the ear traits were significantly correlated with single-plant yield but not with yield per unit area, the single-plant yield and the population ear number jointly form the population yield. Therefore, it is only necessary to study the relationships among the single-plant yield, ear number per unit area, and population yield. This set of relationships indicated that with an increase in density, the ear number of each variety increased substantially while the single-plant yield displayed a downward trend (Fig. 10). When the yield per unit area was highest (i.e., reached the optimal density; see Fig. 10), the ratios between single-plant yield and highest single-plant yield of Zhengdan 958, Xianyu 335, Denghai 661, Yinong 103, and Zhongdan 808 were 0.41, 0.45, 0.48, 0.41, and 0.50, respectively, which all lie in the range of 0.40–0.50. The ear number per unit area of each variety did not have an obvious regular range of changes in values.

#### Response of Dry Matter Accumulation and Distribution to the Density Gradient

**Figure 11:**
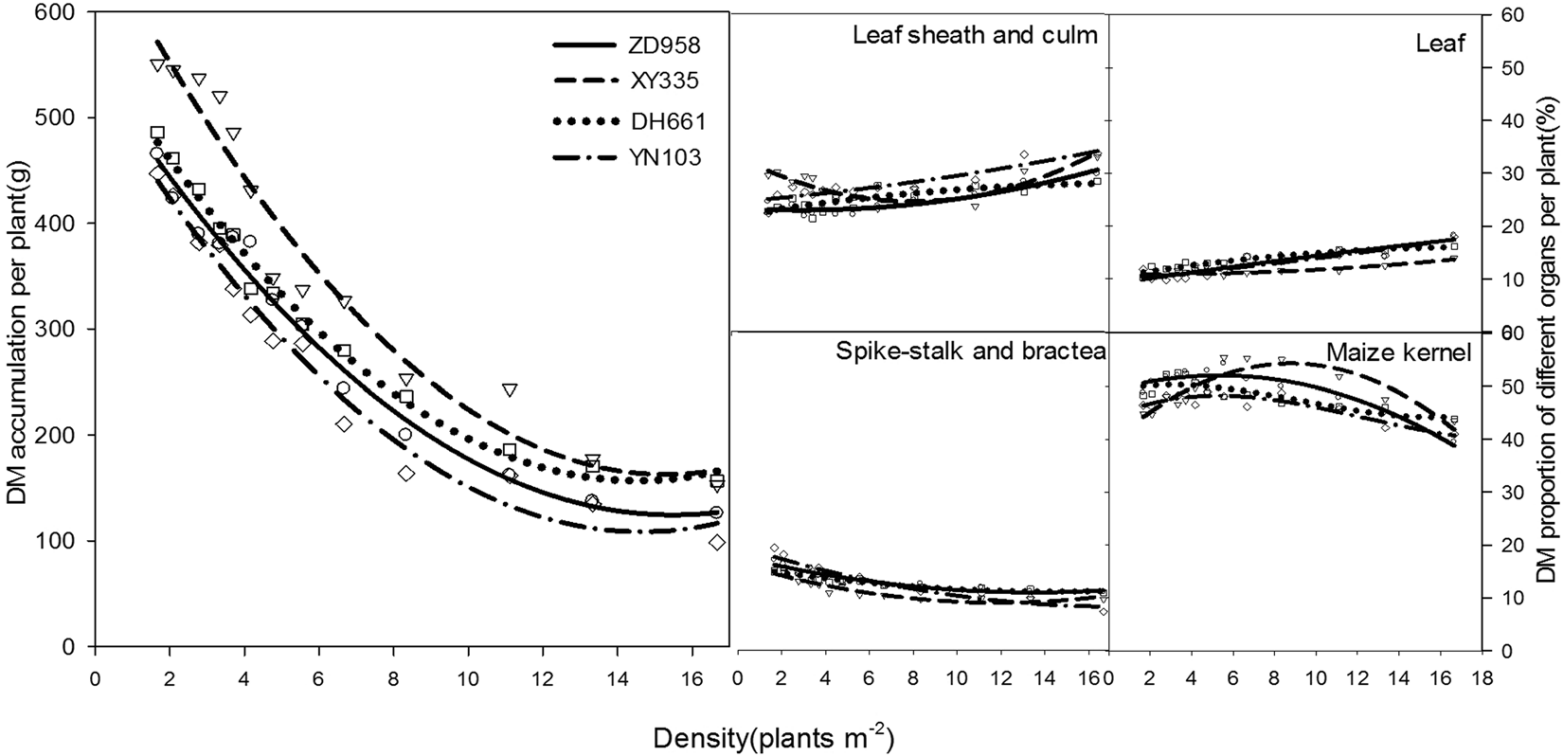
Influence of planting density on dry matter accumulation and the distribution per plant.

With increased planting density, the total dry matter accumulation of a single maize plant at maturity (R6) tended to decline, and the rate of decline was initially high and later low (Fig. 11). The dry matter accumulation of single plants at different planting densities followed the order: Xianyu 335 > Denghai 661 > Zhengdan 958 > Yinong 103. The tall-stalk variety Xianyu 335 had much greater dry matter accumulation than did the short-stalk varieties Denghai 661, Zhengdan 958, and Yinong 103, which indicates that dry matter accumulation is closely related to plant morphological traits.

With changes in planting density, the ranges of the proportions of sheath, leaves, ear-stalk and bracts, and grains were 20–30%, 10–20%, 10–20%, and 35–55%, respectively. The proportion of organs in each variety displayed a similar trend: the percentage of sheath and leaves increased, the percentage of ear stalk and bracts decreased, and the percentage of grain initially rose slightly but later declined. This indicates that with increased density, the proportion of dry matter gradually rose in the nutrition organs but decreased in the reproductive organs.

## Discussion

Maize being an important cereal grain, the crop with highest percentage in production is at the priority in China to enhance production potential and food safety. Modern maize hybrids in China had although been optimized for better tolerance to high planting densities along with higher grain yields^10,17,18^, but still there is lack of a comprehensive method to optimize the planting density in field pertaining to single-plant and population response for yields and morphological characters. Present study has therefore addressed this issue in a very sophisticated manner to quantify the maize varieties’ response against gradually increasing planting densities, and through model simulations. We obtained very concrete results about the density stress tolerance of each studied variety during the five-year period. Hampered grain yields due to the less dry matter accumulation and higher population variability are the key responses to density pressure in maize crop^2,11,16,27^. The most affected traits for reduced grain yields due to increasing density were decreased ear length, ear perimeter, grains per ear and thousand kernels weight. Gradual increase in density has prevented the maize plants gain their genetic potential.

Earlier this was proposed that maize plants has little capacity to generate new reproductive organs at the expense of ample available resources^16,27,28^. Whereas, we found that varieties responded differently in reproducing double ears per plant up to certain increase in plant density, of which the varieties appeared with double sipkes (e.g. Zhengdan 958, Xianyu 335, and Yinong 103) at certain density (ranging from 1.7 to 6.7, 3.7, and 4.8 plants m^−2^, respectively) were unable to reproduce the double ears with a further increase in plant density, sharply. The reason was because the varieties with double ears had higher double-ear rates at lower planting densities due to the lower density pressure. This result is supported by the fact that the density tolerance is purely a genetic function of hybrid, which only yields maximum at certain optimum population density^1,3,6,29,30^. The impact of double-ear occurrence upon the number of grains per ear and thousand-kernel weight was larger than that of density pressure. With equal amounts of nutrition supplied to plants with a double or single ear, the single-ear plants had a different amount of nutrition available per ear. This resulted in a contradiction between the distribution of per plant nutrition and the ear numbers in single-ear plants, which directly resulted in the poor development of average single ear in the double-ear plants compared to the single-ear plants. Therefore, with an increase in planting density, the average rate of occurrence of double ears dropped, and the grains per ear and thousand-kernel weights displayed a transitory upward trend in the lower density range. These results can indirectly be supported by the fact that sink size is mainly determined by the kernel number and its weight, therefore genetic improvements in the grain yields are associated more with the kernel numbers as compared to its size and weight^1,2,31^. However, as the density increased, the rate of occurrence of double ears dropped rapidly. When the rate of occurrence of double ears dropped to a low level, the impact of the ear number per plant on grain number and grain weight was less than that of density pressure. As a result, with increased planting density, the grains per ear and thousand-kernel weight declined, which is an established fact^1,3,10,11,17,32^. As the ear numbers and thousand-kernel weights of the single-ear varieties (Denghai 661 and Zhongdan 808) were affected only by density, they both declined with increased planting density and did not display the trend to increase initially before declining later that was observed in the double-ear varieties.

Maize grain yield is dissected into various physiological processes governing different developmental phases in its life cycle^2^, have two distinct phases overlapping each other, viz. dry matter accumulation (DMA) and the allocation of dry biomass to grain component, thus finally depicting as grain yield. Whereas, “source” components are those affecting DMA and the “sink” components are related to allocation of dry biomass^2,13^. Therefore, the grain yield is finally dependent upon various yield traits which are obviously affected by various biotic and abiotic stresses, of which crowding stress is chronic to maize plants. In our case, much variability occurred for grain yield in varietal response to increasing density stress making the parabolic trends for population yields, while single plant yields showed the linear decrease in grain yields with corresponding increase in plant density. We observed that varieties with more upward leaves (Zhengdan 958, Xianyu 335, Denghai 661) had higher yields as compared to spreading leaves cultivars (Zhongdan 808, Yinong 103), as a function of better light interception at the lower canopy levels producing greater leaf area and higher photosynthesis rates^1,13,32,33^. The tall-stalk variety Xianyu 335 had much greater dry matter accumulation and therefore more yields at population level. Further, it was observed that the ear number per unit area of each variety did not have an obvious regular range of changes in grain yields at gradual density pressure.

Chen *et al*.^33^ had observed that modern Chinese maize hybrids (released after 1990s) that are widely grown in China had highest simulated yield potentials versus older ones. Similarly, Li *et al*.^17^. proposed that Chinese maize hybrids are performing well with higher grain yields under high densities as a function of successful breeding approaches against crowding stress, and the germplasm used for Chinese modern hybrids belongs to decades-old U.S. germplasm. These studies and the other similar ones, however, had not adopted the method, we introduced, to quantify the variety response at population basis to find out the optimum density stress tolerance at gradually increasing rate. Our model simulations have produced the similar results for density resistance as recoded from test and standard plots. Denghai 661 and Xianyu 335 were simulated with high density tolerance, as only these exhibited highest grain yields at densities of 11.1–16.7 plants m^*−*2^. We found that when the planting density was gradually increased from 1.67 plants m^*−*2^ to 16.67 plants m^*−*2^, the yield of each variety had a tendency to initially rise and later fall. Whereas, the adaptability analysis showed that the simulated relative single-plant yields were restored, and plants experienced no density pressure under low-density conditions that produced the highest single-plant yields in the range tested. The potential productivity of individual varieties differed, with the highest single-plant yields in Zhengdan 958. This result is owing to differences in the potential productivity of the various varieties rather than their density-resistance properties. These results were somewhat confirmed by previous studies on similar maize hybrids^10,17,18,33^.

The “*Density-Yield Model*” prediction analysis showed that the density resistance of a variety determined from traditional density-resistance properties coincides exactly with that of the model equation parameters. In addition, the model is simpler and more comprehensive when analyzing changes in the yield of a maize variety and the range of changes in the yields of all varieties. The differences between the varieties and the years was removed by processing the yield data using a normalization technique. The resulting normalized data better simulated the common changes in yield with increasing plant density. Among the simulated curve models, the best simulation effects were achieved for a polynomial fit (1), rational function (2), and Hoerl model (3).

Changes in maize planting density are likely to lead to either an increase or decrease in yield. The degree of influence that density has exerted upon ear traits followed the order maize bald tip, grain number per row, thousand-kernel weight, and ear length. In this study, due to differences in varietal characteristics, the development of single-plant ears responded differently to increasing planting densities. Therefore, we can conclude that the planting density directly affects the single-plant effective ear number of a maize population per unit area. Because density resistance is a comprehensive concept, the entire plant population should be considered in field studies^1,3^.

The single-plant yield directly reflects the response of a maize variety to planting density while the population yield is the result of combined action of the potential productivity of maize and the response to planting density. For plant breeding and to identify density resistance, both factors should be combined to determine whether a maize variety is resistant to high density^17,34^. In this test, the population yields and the single-plant yields are combined to consider the density resistance of a maize variety, and the results are considered more accurate and feasible. We observed that with increased planting density, the yield-increasing effect of population can compensate for the reduction in single-plant yield to some extent. When the compensation is positive, the yield per unit area increases and the increased density is reflected by an increased yield. When the compensation is zero, the yield per unit area reaches a maximum and the planting density for such a yield represents the limit of density resistance for that variety. When the compensation is negative, the population yield decreases and the increased density is reflected by a reduction in yield. In short, the resistance value to the usual effects of high density is based on a slow response of yields to density and low bare-plant rates. In this study, when assessing density resistance and selecting maize varieties, the density resistance and largest yield potential of a variety was comprehensively considered, so as to select the varieties with a higher yield and higher efficiency.

## Conclusion

Several maize varieties were tested against gradually increasing crowding stress for their characteristic response on per plant and on population basis. Density pressure has restrained the studied varieties to gain their genetic potential. We found that gradual increase in density makes a parabolic trend for population yields, while single plant yields decreased in a gradual pattern. Xianyu 335 and Denghai 661 performed best for population yields at high densities (11.1–16.7 plants m^*−*2^), whereas Zhengdan 958 had maximum per plant yield followed by Xianyu 335. Model simulations were also confirmed by actual observations for density resistance, Denghai 661 as best followed by Xianyu 335. The varieties Zhongdan 808 and Yinong 103 were more sensitive to density pressure with maximum rates of change in population yields. Grains per ear and grain weight were found the most affected traits against gradually increased density, with Zhongdan 808 as low-resistant and Zhengdan 958 as high-resistant for this effect. The key features contributing to low per plant yields corresponding to increasing density were decreased ear lengths, ear perimeters, grains per ear and thousand kernels weight. The highest yield performance for these yield traits were exhibited by Xianyu 335 followed by Denghai 661 and Zhengdan 958, under population yields. In conclusion, this study provides a practical approach to quantify the maize varieties for their productivity based on gradually increasing plant density method, which otherwise give inconsistent comparisons between the density-resistance properties of a variety and yield of variety. As we found negligible differences between the standard density plot and gradually-varying density plots, therefore our method is considered more feasible to identify density resistance and variety selection.
